# Comprehensive Analysis of Gene-Environmental Interactions with Temporal Gene Expression Profiles in *Pseudomonas aeruginosa*

**DOI:** 10.1371/journal.pone.0035993

**Published:** 2012-04-27

**Authors:** Kangmin Duan, William M. McCullough, Michael G. Surette, Tony Ware, Jiuzhou Song

**Affiliations:** 1 Department of Oral Biology; Department of Medical Microbiology, University of Manitoba, Winnipeg, Manitoba, Canada; 2 Department of Medicine, Farncombe Family Digestive Health Research Institute, McMaster University, Hamilton, Ontario, Canada; 3 Department of Mathematics and Statistics, University of Calgary, Calgary, Canada; 4 Department of Animal and Avian Science, Animal Science Center, University of Maryland, College Park, Maryland, United States of America; East Carolina University, United States of America

## Abstract

To explore gene-environment interactions, based on temporal gene expression information, we analyzed gene and treatment information intensively and inferred interaction networks accordingly. The main idea is that gene expression reflects the response of genes to environmental factors, assuming that variations of gene expression occur under different conditions. Then we classified experimental conditions into several subgroups based on the similarity of temporal gene expression profiles. This procedure is useful because it allows us to combine diverse gene expression data as they become available, and, especially, allowing us to lay the regulatory relationships on a concrete biological basis. By estimating the activation points, we can visualize the gene behavior, and obtain a consensus gene activation order, and hence describe conditional regulatory relationships. The estimation of activation points and building of synthetic genetic networks may result in important new insights in the ongoing endeavor to understand the complex network of gene regulation.

## Introduction

Current high throughput gene expression techniques, such as oligonucleotide and cDNA microarray, SAGE (series analysis gene expression), promoter array and RNA-seq [1], [2], [3], [4] make it possible to quickly obtain vast amount of time series data in all kinds of organisms under various conditions. Gene expression can be measured simultaneously in a genome-wide manner. Temporal gene expressions under varying environmental conditions have, for instance, been measured during the cell cycle of the yeast *Saccharomyces cerevisiae* and *Bacillus subtilis* [5], [6], [7]. The massively abundant data prove to be invaluable for the possibility of determining the underlying various regulatory relationships among genes and their derivates whereas the inference of genetic interactions remains to be one of the most challenging tasks of modern functional genomics [8], [9], [10], [11], [12], [13].

The biological networks could in principle be divided into several types. The metabolic network is used to denote the network of proteins that synthesize and breakdown cellular molecules. It represents the enzymatic processes within the cell to transform nutrients into energy or into other molecules, i.e. biosynthesis. Protein interaction networks describe communication and signaling networks where the basic reaction is between two proteins or more. The genetic regulatory network is used to represent the general interaction of genes, gene products, and small molecules (i.e. from the DNA level, to the mRNA level, to the protein level). It describes the pathway of gene expression regulation as well as decisions used to turn genes on/off. Deciphering interaction networks is an important task in the post-genomics era.

To build genetic networks, one of the hardest problems is the dimensionality issue, which is the exponential number of potential connections among genes [14], [15]. Current solutions include clustering co-regulated genes via unsupervised analysis [16], [17], [18], [19]. The computing methods involve choosing robust mathematical formalisms for inferring the causal connections between genes etc [20], [21], [22], [23]. Bayesian methods [24] are excellent approaches to infer relationship between genes. They rely on prior information concerning genes, however, and it is difficult to analyze gene expression at the whole genome level due to the number of unknown genes. High throughput gene expression analysis involves many operations and at a not-insignificant cost, consequently there are not many datasets that have measured gene expression levels at a large number of time points. As a consequence, we believe that the current genetic network models generated based on few points provide limited information. Therefore, integrating diverse data types and exploring new ways to construct genetic networks are required. In this paper, to explore the interaction of gene and environmental factors, we assume that gene expression is a comprehensive process of gene and treatments. Because of the interaction, we can classify all experimental conditions into different subgroups based on the similarity of temporal gene expression profiles. Theoretically, these genes within each subgroup showing similar behaviors may share some regulatory mechanism and regulatory network. Finally, by combining all of the information, we estimated a consensus gene activation order within each subgroup. We illustrated our strategy with an example of a 31 gene set in *Pseudomonas aeruginosa*, which was expressed in 72 conditions and measured across 48 time points.

## Results

### The variation of gene expression profile

**Figure 1:**
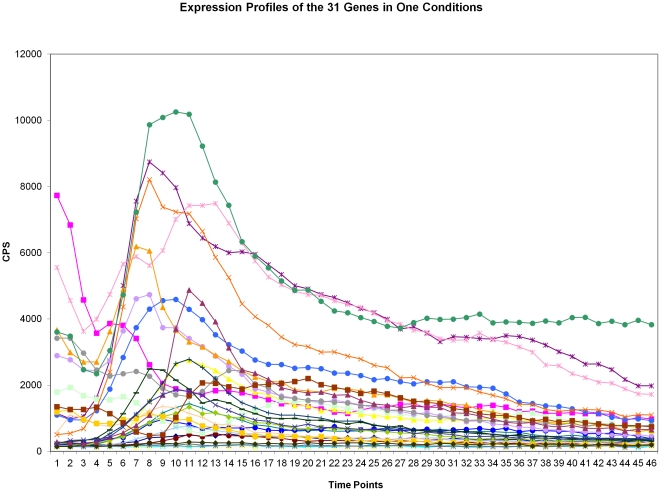
Expression Profiles of the 31 Genes in One Condition.

**Figure 2:**
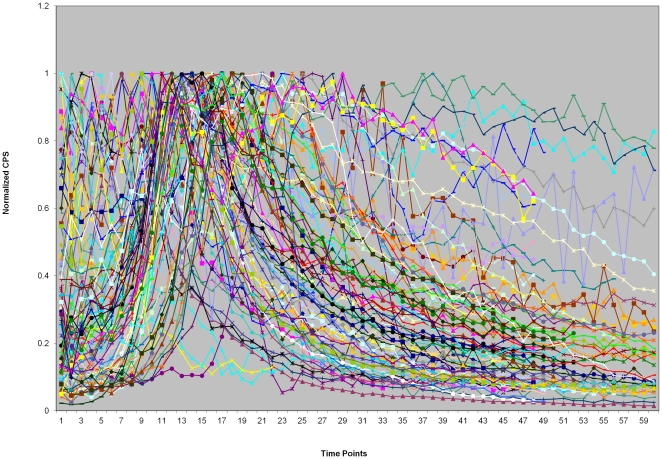
The *aprA* gene expression profiles in 72 conditions and 60 time points.

**Figure 3:**
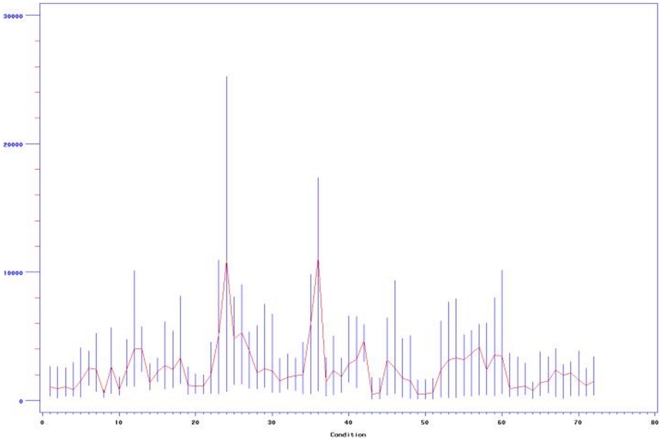
The fluctuation of standard deviation of aprA gene in different conditions and time series.

The large data set was from a unique gene expression experiment of the 31 promoter-reporter set in *Pseudomonas aeruginosa*, tested in 72 conditions, each with 48–60 time points. We first checked the expression profiles of the 31 genes in one condition as shown in Figure 1. We then used the *aprA* promoter-reporter as an example to show the variation in gene expression profiles. The figure shows the expression profile variations of *aprA* in different experimental conditions (Figure 2). From Figure 1 and 2, we can see not only the different behaviors of different genes, but profile differences even for each individual gene under different conditions with the maximum positions shifted among conditions. The profile types increase with condition number. Figure 3 shows the fluctuations of the mean, maximum and minimum for the *aprA* reporter at each time point for all conditions. The results clearly show the expression profiles and levels are condition-specific; they should be classified into several subgroups based on the conditions. An attempt of building a comprehensive genetic network in all conditions is clearly unpractical even though the expression profiles of some genes do not change as dramatically in different treatment conditions as *aprA*. Alternative approaches need to be taken.

### The constructed interaction networks with network motifs

To avoid conflicting gene connections in different experimental conditions and obtain the most popular genetic networks, we clustered all 72 conditions via clustering analysis based on the gene expression profiles (each gene has more than 1400 expression measurements). We used clustering result to guide the formation of environmental condition subgroups, based on the assumption that the condition-dependent expression profiles in each subgroup are similar, and that the genes in each cluster share similar expression pattern and regulatory mechanism. We calculated the transit relationship matrix of the each condition, identified the transit relationship with reference construct pMS402, and then obtained an inferred genetic network for each subgroup.

**Figure 4:**
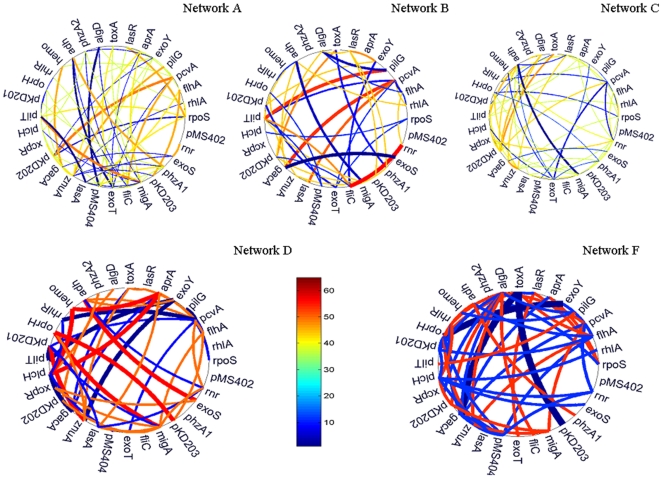
The networks of the five subgroups. The thickness and color of line indicate the popularity in each comprehensive genetic network. The direction of transit is clockwise.

The five constructed interaction networks are shown in Figure 4. The direction of transit relationship is shown by the clockwise turn of the connecting line, and the thickness and color of each connection are proportional to its popularity and strength in the subgroup. The deepest red indicates the strongest positive relationship, otherwise, the dark blue give us the most negative relationship. In the network A-E in Figure 4, we can easily identify the most popular regulation relationships via the thickness and color; for example, *migA* and *pilT* in the network A, gene *rnr* and *fliC* in the network B, *hemo*, *aprA* and *plcH* in the network D, are the most popular positive transit relationships; while gene *adh* and *exoT* in the network A, *adh* and *migA* in network B and C, *pcvA* and *rhlR* in network D and *toxA*, *gacA*, and *PA4350* are the most popular negative relationship in the condition clusters. We also find the transitions are absolutely condition-specific, with the changing of the condition, the direction and strength of the interaction relationship among genes are modified, for example, the relationship between pKD203 and *flhA* in the network A and B is dramatically changed, a mild positive relationship in network A and a negative relationship in network B.

The connections among genes in network A–E (Figure 4) are neither uniformly distributed nor random, similar to that observed with genetic regulatory network motifs [27], [28]. There are a lot of short paths between two genes and highly clustered connections, and several genes have more connections than others. Most of the short paths between two genes are conservative network motifs; these network motifs are very clear in the network architectures. The most popular network motif is analogous a single input module (SIM), i.e., a gene regulates simultaneously several genes; it exists in every network, for example, in network B, gene *gacA* has positive regulation relationship with gene *pvcA* and negative association with *exoS*. The second popular network motif is analogous to dense overlapping regulons (DOR), a set of genes combinatorially control another set of genes, for example in network F, gene *gacA* and *rhlI* (pKD202) coordinately regulate gene *toxA*,. The third common motif is analogous to the feed-forward loop in network B, gene *rpoS* has a mild negative connection with gene *plcH*, but *plcH* has a little stronger negatively feed-back on gene *rpoS*; such a loop is a common motif connection in network B.

### Pattern Matching in Temporal Gene Expression Data

**Figure 5:**
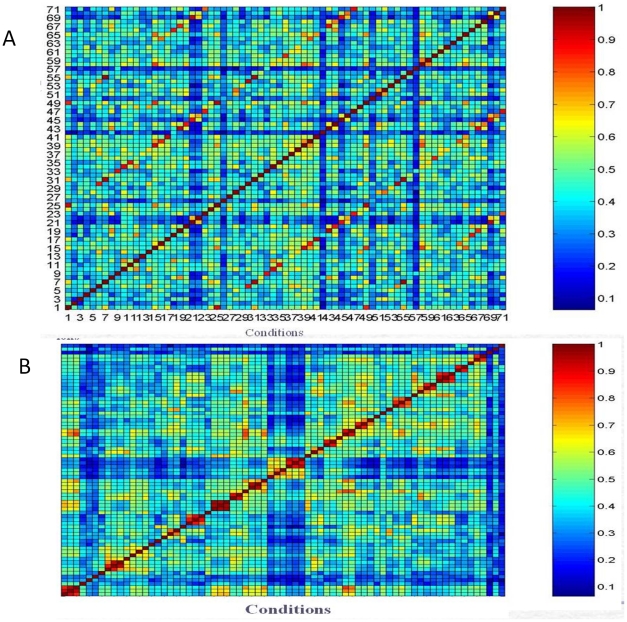
Pattern matching of temporal data. A. This is an original similarity matrix from PCA similarity analysis, the deep red on diagonal is similarity of itself, the similarity is 1. B. This is the reorganized similarity matrix based on clustering analysis.

**Figure 6:**
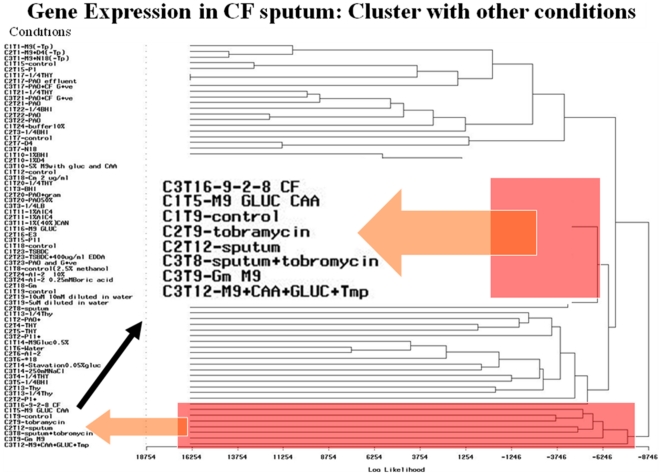
The mapping of unknown condition based on pattern matching of expression. For example, the expression pattern in the complex media of sputum extracts looks most like minimal media growth conditions. The clustering analysis of the expression data for the 72 conditions can yield groups of conditions with similar expression profiles, which can be used for pattern mapping of unknown condition based on expression pattern mapping.

We were also interested in the general pattern matching issue in the temporal dataset. Given an arbitrary set of multivariate temporal data, how can similar patterns be located together? Here we used a novel pattern matching methodology on unsupervised learning and multivariate statistical techniques (Krzanowski 1979). We obtained an original similarity matrix from the PCA similarity analysis, as shown in Figure 5A; the deep red on diagonal is similarity of 1, itself, then the redder the color, the higher the similarity. The reorganized similarity matrix based on clustering analysis is shown in Figure 5B. It illustrates the quality of cluster analysis: the clearer the block, the better the cluster analysis. It is worth noting that the PCA similarity analysis is not only for evaluating the quality of cluster analysis, but also for unknown pattern mapping. For example, the expression pattern in the complex media of sputum extracts looks most like minimal media growth conditions (Figure 6). The clustering analysis of the expression data for the 72 conditions can yield groups of conditions with similar expression profiles, which can be used for pattern mapping of unknown condition based on expression pattern mapping.

### The Determination of Gene Activation Order

**Figure 7:**
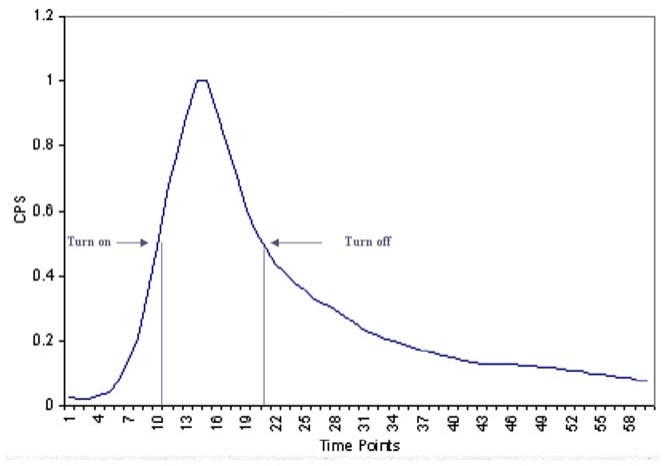
The expression profile of the rpoS gene. The turn point is the half position from lift maximum, the turn off point is the half position from the right maximum.

**Figure 8:**
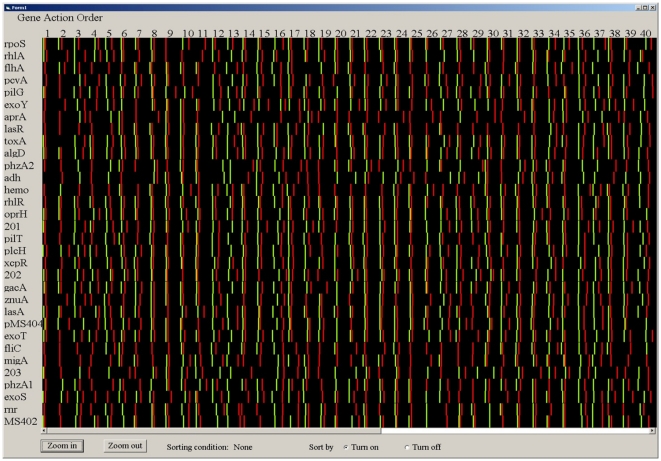
The visualization tool with Visual Basic. The red bar indicates the gene turning off point, the green bar indicates the gene turning on point. The gene order by genes and conditions can obtained via sorting the data with turn on and turn off options.

To understand the mechanism of gene expression and gene activation order, we estimated the gene turning-on and turning-off time points via the least square method, i.e., the half positions of the prior & subsequent of the maximum, as shown in the Figure 7. The visualization tool, as shown in Figure 8, reads and parses the gene expression data into an easily accessible array. The data given were 31 genes in 72 conditions; the green bars and red bars represent the gene turned on and turned off positions, respectively. We were able to sort all the data based on such information and obtain the gene order of turning off and turning on in different networks as shown in Table 1. From the analysis we find that some genes always had the same activity order; they were clearly expressed in stable patterns. For example, gene *lasI*, *rhlI*, *PA4350*, and *znuA* were in quite stable order which was not influenced by different treatment conditions, which is in agreement with the fact that *lasI* and *rhlI* are in a hierarchic order in the bacterium.

**Table 1:** The Gene Activation Order in the Networks.

Network	Status	Activation Order (from left to right)
A	Turn-on	algD, adh, znuA, xcpR/toxA, pKD201/pKD202/pMS404, rhlA, D203, fliC, exoS, exoT
	Turn-off	RpoS, xcpR, migA, znuA, xcpR, pKD201/pKD202/pKD203, rhlA, rhlR, plcH, pMS404, phzA1/phzA2
B	Turn-on	pKD201, pKD203/pKD202, znuA, rpoS/rnr, xcpR, exoS, phzA1/phzA2,
	Turn-off	phzA1/xcpR, pKD201/pKD202/pKD203, rpoS/rnr, phzA1/phzA2, hemo, exoY, exoS.
C	Turn-on	Adh/aprA, exoY, toxA/xcpR/rpoS, pKD201/pKD202/pKD203, plcH, pilG, phzA1/phzA2, lasR, znuA, rnr
	Turn-off	plcH, rhlA/rhlR/toxA, pKD202/pKD201, exoS/lasR, rpoS, algD, phzA1/PhzA2, migA, pMS404, znuA, aprA, pKD203,
D	Turn-on	pKD202, pKD201, znuA, pKD203,
	Turn-off	PlcH, rpoS, pKD201, pKD202, pKD203, rnr, xcpR, toxA, algD, lasA, pilT
E	Turn-on	pMS404, rhlR, toxA
	Turn-off	pMS404, znuA, lasR, phzA1

## Discussion

The main purpose of exploring gene-environmental interaction is to provide indications about regulation mechanisms of the genes in response of environmental changes. The abundance of genome sequences and high throughput gene expression data is providing input for reverse engineering of genetic networks [29], [30], [31], [32], [33], [34], [35]. The critical issue is how to use all kinds of information which include both genome and gene expression information to infer the relationships between genes and their activities. The strategy presented here offers new capability of extracting fundamental interaction network from expression data, and identifying the most popular regulatory relationships and gene activity order.

The most powerful feature of our strategy is the intensive temporal gene expression profile information. Because gene expression varies with the conditions, the process of building an interaction network is a difficult one. We assume that there should be a virtual network in the cell process, the connection among genes should be stable no matter observable or not. Diverse experimental conditions may enhance or repress transcription from DNA to mRNA, and lead to the changes in mRNA levels in different experimental conditions. In the past, many gene regulatory studies involved only a limited number of genes in some given conditions. It is difficult to infer genetic regulatory networks or predict connections among genes when using Bayesian theory and advanced data mining method for large and diverse prior information or expression data. Here, our key step is to cluster all conditions into several subgroups, where each of the genes in the sub-cluster set has similar expression profile and thus may share a common biochemical regulatory mechanism. Hence, the synthetic regulatory networks built are for specific condition clusters. The most popular connections indicate the fundamental regulatory relationship in each specific condition subgroup. The main advantages of the method are simplifying the data by grouping and potentially avoiding the confliction of regulatory relationship in information combination.

Setting a reference gene is the second feature of our strategy. The threshold of genetic connections is a critical issue; it is related to the confidence and quality of the genetic regulatory network. In our research, pMS402 should not have any regulatory relationships with other genes. We used it as a control, and only picked up genes with stronger coefficients than that of pMS402. This procedure set a biological basis for the relationship matrix in each network. In future research, a statistical significance testing method will be provided for the regulations. In the predicted interaction networks, we also observed motifs analogous to the feed forward loop, single input module (SIM) and dense overlapping regulons (DOR) [27], [36]. The network motifs are important for allowing multiple steady states of gene expression rates, and maintaining homeostasis of gene expression rates. Gene networks incorporated with loops, modules and regulons can readily produce oscillations and even more complex behaviors, such as quasiperiodic or chaotic variations in gene transcription rates [37].

The measuring of the turning on and turning off points via the half of maximum expression is another merit of the analysis strategy. Our strategy estimates the two time points, and pools all gene expression data in all conditions. It reveals pronounced gene activation asymmetries, which emphasizes that gene expression during growth of bacteria is overall a strongly constrained and ordered process, and exposes the activation order of stable genes and environmental sensitive genes. The expression time courses analysis could reveal physiology state transitions in response to different environmental conditions if we have many conditions and enough time point measurements.

Overall, our computational framework adopts the principal idea that the gene expression level is the outcome of genetic regulations under specific experimental conditions, which allows classifying all experimental conditions into different subgroups based on their expression profiles, and combing more diverse gene expression data sets. The pattern matching methodology is generally applicable to a wide variety of pattern matching problems, including abnormal gene expression analysis, unknown pattern mapping and evaluation of temporal gene expression data. In addition, the estimation of activation points provides a new tool to understand the complex network of gene regulation.

## Materials and Methods

### Gene expression data

Promoter-reporter (*luxCDABE*) fusions for selected *P. aeruginosa* genes previously constructed [25], [26] were used in this study. These genes are currently known virulence factors and genes that are associated with pathogenicity in *P. aeruginosa*. Reporters used in this study are listed in Table 2. Briefly, the promoter regions of selected *P. aeruginosa* virulence factors were amplified by PCR using oligonucleotide primers synthesized according to the PAO1 genome data and PAO1 chromosomal DNA as the template. The PCR amplified promoter regions were then cloned into the *Xho*I-*Bam*HI sites of pMS402 and transformed into PAO1 by electroporation. PCR, DNA manipulation and transformation were performed following general procedures. The promoterless *luxCDABE* operon in pMS402 enables the activity of the promoter fused upstream of the operon to be measured as counts per second (cps) of light production in a Victor^2^ Multilabel Counter.

**Table 2:** The list of the gene reporters used in this study.

Gene	Function	PA number
lasI (pKD201)	AHL synthase	PA1432
lasR	AHL dependent transcriptional regulator	PA1430
rhlI (pKD202)	AHL synthase (rhlL)	PA3476
rhlR	AHL dependent transcriptional regulator	PA3477
lasA	protease (staphylolytic protease preproenzyme LasA)	PA1871
lasB (pMS404)	Elastase	PA3724
aprA	alkaline protease (alkaline metalloproteinase precursor)	PA1249
xcpP	xcp secretion pathway (differient orientation from xcpR)	PA3104
xcpR	xcp (general secretion pathway protein E)	PA3103
rhlA	rhaminolipid (rhamnosyltransferase chain A)	PA3479
rpoS	stationary phase sigma	PA3622
gacA	transcriptional activator,response regulator	PA2586
pilT	Type IV pili (twitching motility protein PilT, pilT I followed by pilU)	PA0395
pilG	Type IV fimbrial (Part of the pilGHIJKL gene cluster)	PA0408
algD	alginate (GDP-mannose 6-dehydrogenase AlgD), first of 18-kb alginate operon.	PA3540
plcH	hemolytic phopholipaseC (hemolysin) precursor	PA0844
toxA	exotoxinA	PA1148
exoS	exoenzymeS (ADP-ribosyltransferase)	PA3841
exoT	exoenzymeT (99% similar to ADP-ribosyltransferase (exoenzyme 53))	PA0044
exoY	adenylate cyclase	PA2191
PhzA1	pyocyanin synthesis (phenazine synthesis cluster)	PA4210
PhzA2	pyocyanin synthesis (phenazine synthesis cluster,first gene)	PA1899
pvcA	pyoverdine biosynthesis protein PvcA, first of four ORF cluster	PA2254
hem	putative hemagglutinins (43% identity to B. pertussis)	PA0041
rnr	exoribonuclease RNase R (virulence protein VacB) class2	PA4937
adh	probable adhesion protein	PA2407
znuA	probable adhesion	PA5498
fliC	flagellin	PA1092
flhA	flagellar biosynthesis protein	PA1452
migA	probable glycosyl transferase (mucin-inducible gene)	PA0705
oprH	PhoP/Q and low Mg2+ inducible outer membrane protein H1 precursor	PA1178
PA4350 (pKD203)	Putative hemolysin	PA4350

Initial cultures were grown in M9 minimal medium supplemented with casamino acid (0.1%), and glucose (0.5%) with trimethoprim added at 200 µg/ml. Overnight cultures of the reporter strains were diluted 1∶200 in a 96-well microtiter plate and the promoter activity of the virulence factors in different conditions was measured every 30 minutes for 24 hours. Bacterial growth was monitored at the same time by measuring the optical density at 620 nm (OD_620_) in the Victor^2^ Multilabel Counter. All the expression assays were carried out at least twice. Growth conditions examined are listed in Table 3. These conditions include different growth media that are frequently used in the microbiology laboratories and conditions containing factors found in *P. aeruginosa* infection sites e.g. sputum extract from cystic fibrosis patients.

**Table 3:** The environmental conditions tested.

Condition number	Condition code	Description	Condition number	Condition code	Description
1	C1T1	M9 medium (-Tp)	37	C2T13	THY medium
2	C1T2	with PAO spent culture	38	C2T14	stavation M9+0.05%gluc
3	C1T3	BHI medium	39	C2T15	co-culture with P1 isolate
4	C1T4	LB medium	40	C2T16	co-culture with E3 isolate
5	C1T5	M9+0.05% CAA	41	C2T17	with 1% PAO biofilm effluent
6	C1T6	1% H2O	42	C2T18	with subinhibitory Gm
7	C1T7	control 1	43	C2T19	with 10 uM AI-2
8	C1T8	with 2.5% methanol	44	C2T20	with supernatant of PAO+gram
9	C1T9	control 2	45	C2T21	with supernatant of PAO
10	C1T10	M9+1% BHI	46	C2T22	with supernatant of PAO
11	C1T11	M9 with 1% AHL C4	47	C2T23	TSBDC+400 ug/ml EDDA
12	C1T12	control 3	48	C2T24	with AI-2
13	C1T13	LB medium	49	C3T1	co-culture with isolate N18(-Tp)
14	C1T14	M9 with 0.5% Gluc	50	C3T2	co-culture with isolate P11
15	C1T15	control 4	51	C3T3	1/4 diluted LB
16	C1T16	M9+1% Gluc	52	C3T4	1/4diluted THY
17	C1T17	1/4 diluted THY	53	C3T5	1/4 diluted BHI
18	C1T18	control 5	54	C3T6	with AI-2 analog #18
19	C1T19	control 6	55	C3T7	co-culture with isolate N18
20	C1T20	1/4 diluted THY	56	C3T8	with sputum extract and tobromycin
21	C1T21	1/4 dilute THY	57	C3T9	with subinhibitory Gm
22	C1T22	1/4 diluted BHI	58	C3T10	M9 with 5% gluc and CAA
23	C1T23	TSBDC medium	59	C3T11	1%(40% CAN
24	C1T24	PBS buffer 10%	60	C3T12	M9+CAA+Gluc+Tmp
25	C2T1	co-culture with isolate D4(-Tp)	61	C3T13	1/4 diluted THY
26	C2T2	co-culture with isolate P1	62	C3T14	250 mM NaCI
27	C2T3	1/4 diluted BHI	63	C3T15	co-culture with isolate P11
28	C2T4	THY medium	64	C3T16	co-culture with isolate 9-2-8
29	C2T5	THY medium	65	C3T17	with supernatant of PAO+CF G+ve isolate
30	C2T6	with 25uM AI-2	66	C3T18	with Cm 2 ug/ml
31	C2T7	co-culture with isolate D4	67	C3T19	5 uM AI-2 diluted in water
32	C2T8	with 1% sputum extract	68	C3T20	with supernatant of PAO 50%
33	C2T9	with subinhibitory tobramycin	69	C3T21	with supernatant of PAO+CF G+ve isolate
34	C2T10	1% D4 supernatant	70	C3T22	with supernatant of PAO
35	C2T11	1% AHL C4	71	C3T23	with supernatant of PAO and G+ve isolate
36	C2T12	with 2.5% sputum extract	72	C3T24	0.25 mM Boric acid

### Gene expression level model

In gene expression analysis, we assume that any gene expression level is a comprehensive result of gene effects and environmental effects. The simple formula is as follows:
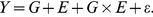
(1)Here, 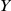 is the column vector of expression level measurements of m genes in a specific treatment 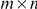 matrix if there are n measurements at n time points; 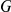 is the gene effect. The effects could be single gene effects, or interaction effects among multiple genes, or a complicated genetic regulatory network for a set of genes or whole genes in a genome. The effects indicate the inner biochemical and physiological mechanisms; 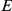 is the environmental effect, it represents effects of different experimental treatments; 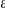 is a random error.

### Clustering analysis of gene expression with different conditions

The large scale data consisted of gene expression measurements in 72 conditions over 48 time points, all measurements were corrected with OD value, and normalized in each condition. The distance between two clusters is defined by
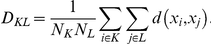
If 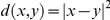, then
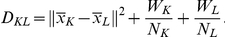
The combinational formula is
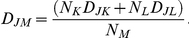
In average linkage the distance between two clusters is the average distance between pairs of observations, one in each cluster. Average linkage tends to join clusters with small variances, and is slightly biased toward producing clusters with the same variance.

### Analysis methods

We deal with gene expression data from m genes over n time points 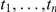. The data are represented in an 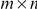 array Y, and we assume that the gene expression levels at time 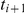 are determined by the expression levels at time 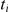 via the functional
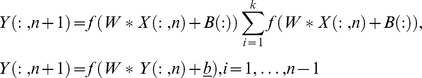
where W is an 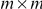 matrix, 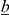 is a 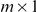 vector, f is some nonlinear switching function (for example a sigmoid centered at 0) which acts on each element of the 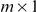 vector 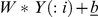 to produce the 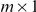 vector 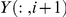 The notation used here has the following meaning: if A is an array, then 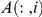 denotes the 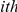 column of A.

Equation above can be partially inverted to give
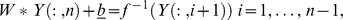
and we are trying to use these equation to determine the matrix W and the vector 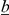. To do this most conveniently, we group the equations together, writing: 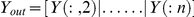 and 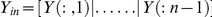 (note that these are 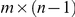 arrays). We also write
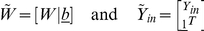
where 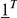 is a row of n-1 ones. Then 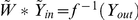, and we want to solve this system for 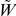. This is done (in a least-squares sense if the system is over or under-determined) by using the pseudoinverse of 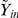.

### The thresholds of transit relationship matrix

Because pMS402 contains the promoterless *luxCDABE* operon, we set pMS402 as a reference. Theoretically, it does not have any relationships with other genes. To avoid meaningless regulation associations among the gene set, we took the absolute value of the coefficients of the gene pMS402 among the relationship matrix to be the threshold in each condition. If coefficients among two genes in the transit relationship matrix are greater than the threshold, the coefficient is positive, and is given the value 1, indicating positive transit relation, otherwise it is given the value −1, representing a negative transit relationship; any coefficient less than the threshold is given the value 0, representing no regulatory associations between the two genes.

### Pattern similarity analysis

Assuming that two data sets contain the same n variables but not necessarily the same number of measurements, for each data set we consider a principal component analysis (PCA) model containing k principal components (PC). The PC number, k, is chosen such that k principal components explain at least 95% of the total variance of each data sets. The similarity between the two data sets is measured by comparing their principal components, defining as a single number. Let be the PC number describing at least 95% of the variance in data set S, and let as the PC number in data set H, which also describe 95% of its variance. If 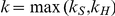, it ensures that k principal components explain 95% of the variance in each data set. The PCA similarity factor compares can be calculated from the angels between principal components:
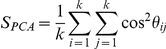
where *θ_ij_* is the angle between the principal component i in data set S and principal component i in data set H. The similarity factor can also be expressed as
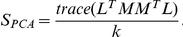
here, L and M contain the k most significant principal components for S and H.

### Visualization of gene turning-on and turning-off position

To describe gene effects mechanisms in regulatory networks, we defined expression prior to the maximum as the turning on section, with the half position of the maximum being the gene turning on point. Expression subsequent to the maximum is the gene turning off section, with the half position of the maximum being the gene turning off point. We estimated the two positions via the least squares method. This visualization program reads and parses the gene expression data into an easily accessible array, and was created using Visual Basic. The data given were 31 genes in 72 conditions for 48 time points. When the gene was turned on there would be a 1, when the gene was turned off there would be a −1 and the rest of the time points would be zero. The screen was divided into 40 sections, where each section represented a particular condition. The section is divided into 60 different areas. When the gene was turned on, a green bar would be placed in one of these 60 areas, and when the gene was turned off a red bar would be placed into one of these areas. It was not unusual for a gene to be on before the experiment or on after the experiment was done, and in this case the appropriate bar was removed. The program also allowed you to limit the number of conditions that was displayed at any one time. For ease of viewing the upper limit was 40 conditions, and for ease of coding, the lower limit was put at 5. The display can be sorted four ways. The first is by the order of activation in one condition, or sorting by which gene was on first. The second is by which gene turned off first in a particular condition. The third is sorting the conditions based upon which one turned on a specific gene first. The last is sorting the conditions based upon which condition turned off a specific gene first.
